# Tranexamic acid combined with fluid gelatin in perioperative blood loss management of total hip arthroplasty for elderly femoral neck fractures: a single-center retrospective analysis

**DOI:** 10.3389/fsurg.2026.1728145

**Published:** 2026-02-27

**Authors:** Chao Zhao, Bobin Fu, Longyun Li, Shaowei Sun, Lifu Wang, Cong Xiao

**Affiliations:** 1 North Sichuan Medical College, Nanchong, China; 2 Department of Orthopedics, The Third Hospital of Mianyang Sichuan Mental Health Center, Mianyang, China

**Keywords:** femoral neck fracture, fluid gelatin, hidden blood loss, perioperative blood loss, total hip arthroplasty, tranexamic acid

## Abstract

**Objective:**

Tranexamic acid (TXA) and fluid gelatin (FG) are widely used to reduce perioperative blood loss in total hip arthroplasty (THA). However, the efficacy of single-method hemostatic strategies is increasingly insufficient for meeting clinical demands. The aim of this study was to evaluate the efficacy of TXA in combination with FG for perioperative blood management.

**Methods:**

This retrospective study enrolled 301 patients with unilateral femoral neck fractures who underwent minimally invasive piriformis approach total hip arthroplasty (between 2019 and 2023) and received hemostatics. Patients were divided into TXA (*n* = 93), FG (*n* = 102), and TXA + FG (*n* = 106) groups. A control group (*n* = 107) with the same inclusion/exclusion criteria was selected. Collected data included demographic characteristics, fracture classification, perioperative clinical parameters, and laboratory findings. The primary and secondary outcome measures included total blood loss (TBL), visible blood loss, hidden blood loss, postoperative hemoglobin and hematocrit levels, blood transfusion, and postoperative complications.

**Results:**

The clinical results of 408 patients indicated that the combination of TXA and FG significantly reduced perioperative blood loss in THA via MIS-TPA (550.68 ± 327.61 mL in the TXA + FG group, 732.50 ± 362.84 mL in the TXA group, 817.19 ± 375.46 mL in the FG group, and 982.99 ± 428.81 mL in the control group; *p* < 0.001) without an increase in thromboembolic events or wound-related complications.

**Conclusion:**

Combined intravenous TXA and intra-articular FG administration provided superior perioperative blood loss control and did not increase the rate of complications.

## Introduction

With the accelerating global aging process, the incidence of femoral neck fractures continues to escalate, leading to a progressively increasing volume of total hip arthroplasty (THA) procedures performed annually (1). THA is widely recognized as the preferred therapeutic option for unstable femoral neck fractures in elderly patients, offering notable advantages including rapid postoperative recovery and substantial functional improvement (2). However, the procedure is associated with several perioperative hazards, particularly blood-related complications. The traditional posterolateral approach, a classic technique for THA, has long been utilized clinically owing to its adequate surgical exposure and broad operative field. Nevertheless, this approach results in greater soft tissue trauma and relatively increased perioperative bleeding—an issue of particular concern in elderly patients, as it can induce anemia, delay rehabilitation, and even adversely affect postoperative outcomes (3).

To mitigate the risk of perioperative blood loss, minimally invasive surgical approaches (4, 5) and the antifibrinolytic agent tranexamic acid (TXA) (6, 7) have been routinely used in clinical practice in recent years to reduce overt bleeding to a certain extent. However, several studies have shown that even after the standardized application of TXA, elderly patients still experience about 600 mL of hidden blood loss during and after THA, which is difficult to assess and manage in a timely manner, posing a potential threat (8) to the elderly population with limited cardiopulmonary reserve. Therefore, optimizing intraoperative hemostasis strategies and reducing hidden blood loss remain key clinical concerns. Relevant studies (9) have shown that fluid gelatin (FG) has a good hemostatic effect locally and potential adjuvant hemostatic value. Our previous work also demonstrated that fluid gelatin has a certain hemostatic effect (10). Therefore, the present study systematically compares the effects of different approaches using fluid gelatin in combination with TXA during total hip arthroplasty for elderly patients with femoral neck fractures, aiming to explore better perioperative bleeding control strategies and provide evidence-based support for safe surgical practice in the elderly population.

## Methods

### Study design

This retrospective study analyzed patients who underwent total hip arthroplasty due to unilateral femoral neck fractures at our hospital between January 2019 and December 2023. The ethical committee of our institution approved the study protocol (No.2023/59).

### Inclusion criteria

(1) age >60 years; (2) clear imaging diagnosis of a fresh unilateral femoral neck fracture; and (3) proposed primary total hip arthroplasty with minimally invasive piriformis approach.

### Exclusion criteria

(1) open or old fractures; (2) pathological fractures; (3) multiple traumas or fractures at other sites; (4) preoperative history of thromboembolism, such as deep vein thrombosis, cerebral infarction, and pulmonary embolism; (5) severe liver or kidney dysfunction and abnormal coagulation function; (6) use of anticoagulants 1 week prior to surgery; (7) hemorrhagic disorders or other blood system disorders; (8) surgery requiring a 24-h intravenous infusion volume exceeding 2,000 mL; (9) history of allergy to TXA or FG; and (10) incomplete clinical data.

After applying the exclusion criteria, 301 patients who received treatment with hemostatic drugs were included in the study group. According to the application of hemostatic agents during the perioperative period, they were divided into the following three groups: the TXA group (intravenous TXA only; *n* = 93), the FG group (topical application of FG within the joint cavity only; *n* = 102), and the TXA + FG group (combined intravenous TXA and topical FG; *n* = 106).

A control group of 107 was retrospectively selected from patients who underwent total hip arthroplasty due to unilateral femoral neck fractures at our hospital during the same period and met the same inclusion and exclusion criteria.

### Perioperative management

All patients were evaluated by the anesthesiology department prior to surgery and administered general anesthesia. During the operation, patients were placed in the healthy lateral position and routinely disinfected with bandings. The operation was performed by the same experienced joint surgery team.

### Surgical approach

In the minimally invasive group, the surgical approach uniformly adopted the minimally invasive trans-piriformis muscle approach (11, 12). The specific procedure involved making a surgical incision approximately 9.0 cm in length along the direction of the gluteus maximus muscle fibers, with the direction of the surgical incision forming an angle of approximately 30° with the long axis of the femoral shaft. The skin and subcutaneous tissue were cut in sequence, the gluteus maximus fibers were blunted and separated, the gluteus medius was pulled toward the proximal end, and the hip joint of the affected limb was flexed and internally rotated to expose the piriformis insertion point. The absorbable suture mark and the piriformis insertion point were placed above the external rotation muscle group for protection, and the exposed and “U”-shaped joint capsule was placed above and below the femoral neck. The lesser trochanter is identified by palpation, and the femoral neck is osteotomized perpendicularly at a distance of 0.5 cm proximal to its most prominent point to excise the femoral head. The acetabulum was routinely prepared and the globular cup was implanted with screws and polyethylene lining. After bone marrow expansion on the femoral side, a femoral stem prosthesis was implanted. A ball head was fitted, hip reduction was completed, and the stability of the prosthesis was dynamically evaluated during the operation. The joint capsule was sutured, the piriformis insertion point was reconstructed, and intradermal suturing of the wound was performed. No drainage tube was placed during the procedure.

### The use of hemostatic drugs

The control group: Neither TXA nor FG was administered during the procedure.

The TXA group: A total of 1 g of TXA (Kainaiting, Brilliant Pharmaceutical, Chengdu, China) was administered intravenously 15 min prior to surgery, followed by an additional 1 g administered 3 h postoperatively, in accordance with the protocol recommended in the *Expert Consensus on Tranexamic Acid and Anticoagulant Use in the Perioperative Period of Enhanced Recovery After Orthopedic Surgery in China* (7).

The FG group: A fluid gelatin injection (Surgiflo, Ethicon, Cincinnati, USA) was locally injected into the joint cavity immediately after complete closure of the joint capsule and before wound suturing. The total injection volume was standardized at 10 mL, which was evenly distributed over the surgical field (including the acetabular fossa, femoral medullary cavity, and soft tissue dissection area) by the operating surgeon to ensure coverage of potential bleeding sites. The fluid gelatin exhibited favorable biocompatibility and diffusion characteristics, allowing uniform distribution within the joint capsule and maintaining local hemostatic efficacy for 4–6 h without interfering with tissue healing or prosthesis integration.

The TXA + FG group: TXA and FG were used in combination. TXA and FG were administered using the same above-mentioned regimen.

### Postoperative anticoagulation and blood transfusion management

All patients received routine postoperative anticoagulation to prevent deep vein thrombosis. Enoxaparin (Clexane, Sanofi-Aventis, Maisons-Alfort, France) was administered subcutaneously at a dose of 4,000 IU once daily, starting 6 h after surgery and continued for 5–7 days. After discharge, patients received oral rivaroxaban (Xarelto, Bayer, Leverkusen, Germany) at 10 mg once daily for a total duration of 14–35 days, with the precise course individualized based on each patient's thromboembolic risk assessment.

Perioperative blood transfusion management followed standardized transfusion principles. Red blood cell transfusion was indicated for patients with hemoglobin (HGB) levels <70 g/L. For patients with HGB levels between 70 and 100 g/L, transfusion decisions were based on clinical manifestations of anemia—such as fatigue, chest tightness, or dyspnea. In principle, transfusion was not recommended for patients with HGB levels >100 g/L.

### Outcomes measurement

#### Patient demographics

The demographics of patients in all groups were collected, including gender, age, body mass index (BMI), initial diagnosis, and American Society of Anesthesiologists (ASA) classification score.

#### Perioperative blood loss

Blood loss parameters—including total blood loss (TBL), hidden blood loss (HBL), and visible blood loss (VBL)—as well as perioperative hemoglobin and hematocrit levels, together with the number and volume of blood transfusions, were collected and analyzed.

The calculation procedures for TBL, HBL, and VBL are outlined in the following.

Nadler's formula (13) was used to estimate the patient blood volume (PBV):

$$\mathrm{PBV}\;(L)\;=\;\mathrm{height}\;\left(m^{3}\right)\;\;\times \;0.367\;+\;\mathrm{weight}\;(\mathrm{kg})\;\;\times \;0.032\;+\;0.604\;(\mathrm{for}\;\mathrm{male}\;\mathrm{patient})$$

$$\mathrm{PBV}\;(L)\;=\;\mathrm{height}\;(m^{3})\;\;\times \;0.356\;+\;\mathrm{weight}\;(\mathrm{kg})\;\;\times \;0.033\;+\;0.183\;(\mathrm{for}\;\mathrm{female}\;\mathrm{patient})$$

Gross' formula (13) was used to calculate TBL based on PBV and hematocrit (HCT) drop:

$$\mathrm{TBL}\;(\mathrm{mL})\;=\;\mathrm{PBV}\;(L)\;\;\times \;(\mathrm{HC}T_{\mathrm{pre}}\text{-}\mathrm{HC}T_{\mathrm{post}})/\mathrm{HC}T_{\mathrm{ave}}\;\times 1,000$$

where HCTpre is the preoperative HCT on the day before surgery, HCTpost is the HCT on the third postoperative day, and HCTave is the average of HCTpre and HCTpost.

The VBL was estimated based on the amount of liquid in the negative pressure drainage bottle, the amount of liquid in the gauze, the amount of saline and postoperative drainage volume. A piece of gauze was soaked with approximately 20 mL of liquid. The following formula was applied:

VBL (mL) = liquid in the negative pressure drainage bottle + the amount of liquid in the gauze – the amount of saline + postoperative drainage volume (14).

HBL was calculated as follows: HBL (mL) = TBL (mL) + blood infusion (mL)  – VBL (mL). Moreover, one unit of red blood cells was recorded as equivalent to 200 mL (15).

The types and volumes of perioperative blood transfusions were also systematically documented. In addition, the volume of intravenous fluid administered during the perioperative period was also recorded.

#### Perioperative complications

Postoperative complications occurring within 90 days were recorded, including deep vein thrombosis (DVT), pulmonary embolism (PE), infection, and bruising. PE was diagnosed using computed tomography pulmonary angiography, and DVT was diagnosed using duplex ultrasound scanning. Each patient received a postoperative lower limb venous ultrasound examination. Computed tomography pulmonary angiography was performed for patients with symptomatic PE.

#### Coagulation function

The coagulation function of the patients was assessed on the third day after the operation. Parameters collected included activated partial thromboplastin time (APTT), prothrombin time (PT), D-dimer level, and fibrinogen (FDP) level.

### Statistical analysis

Continuous data were presented as the mean ± standard deviation and were compared using an analysis of variance, with Tukey’s honestly significant difference tests applied for pairwise comparisons. Categorical data were presented as counts and percentages, and differences among groups were assessed using Chi-square or Kruskal–Wallis tests, with Bonferroni correction applied for multiple comparisons. All analyses were conducted using SPSS (version 22.0, IBM Corp., Armonk, NY, USA).

## Results

### Patient demographics

There were no statistically significant differences the four groups of patients in terms of gender, age, height, weight, BMI, surgical site, fracture classification, ASA grade, operation time, incision length, preoperative PT, and APTT, as shown in Table 1.

**Table 1:** Baseline characteristics of the patients*.

Characteristics	Control group (*n* = 107)	TXA group (*n* = 93)	FG group (*n* = 102)	TXA + FG group (*n* = 106)	*P*-value
Demographic information					
Sex (M/F) (no. of patients, %)	54/53 (50.5%/49.5%)	47/46 (50.5%/49.5%)	51/51 (50.0%/50.0%)	50/56 (47.2%/52.8%)	0.869
Age (year)	67.54 ± 2.84	67.24 ± 2.96	67.95 ± 3.06	67.75 ± 2.88	0.363
Height (m)	1.65 ± 0.04	1.64 ± 0.05	1.63 ± 0.07	1.65 ± 0.06	0.542
Weight (kg)	61.3 ± 5.8	60.9 ± 6.2	61.1 ± 5.5	60.7 ± 6.0	0.834
BMI (kg/m^2^)	22.47 ± 2.44	22.62 ± 2.79	22.66 ± 2.63	22.59 ± 3.11	0.962
Surgical site (L/R) (no. of patients, %)	62/45 (58.0%/42.0%)	44/49 (47.3%/52.7%)	59/43 (57.8%/42.2%)	48/58 (45.3%/54.7%)	0.131
Garden classification (no. of patients, %)					
I	7 (6.5%)	5 (5.4%)	6 (5.9%)	7 (6.6%)	1.000
II	38 (35.5%)	31 (33.3%)	35 (34.3%)	36 (34.0%)	
III	45 (42.1%)	40 (43.0%)	43 (42.2%)	44 (41.5%)	
IV	17 (15.9%)	17 (18.3%)	18 (17.6%)	19 (17.9%)	
ASA Class (no. of patients, %)					
I	7 (6.5%)	9 (9.7%)	6 (5.9%)	14 (13.2%)	0.549
II	55 (51.4%)	48 (51.6%)	53 (52.0%)	55 (51.9%)	
III	45 (42.1%)	36 (38.7%)	43 (42.2%)	37 (35.0%)	
Comorbidities (no. of patients, %)					
Hypertension (yes/no)	26 (24.3%)/81 (75.7%)	22 (23.7%)/71 (76.3%)	24 (23.5%)/78 (76.5%)	25 (23.6%)/81 (76.4%)	0.999
Diabetes (yes/no)	21 (19.6%)/86 (80.4%)	18 (19.4%)/75 (80.6%)	20 (19.6%)/82 (80.4%)	25 (23.6%)/81 (76.4%)	0.852
Smoking history (yes/no)	35 (32.7%)/72 (67.3%)	33 (35.5%)/60 (64.5%)	37 (36.3%)/65 (63.7%)	37 (35.0%)/69 (65.1%)	0.956
Drinking history (yes/no)	95 (88.8%)/12 (11.2%)	83 (89.2%)/10 (10.8%)	91 (89.2%)/11 (10.8%)	95 (89.6%)/11 (10.4%)	0.998
Preoperative laboratory values					
Preoperative PT (s)	11.25 ± 0.85	11.15 ± 0.90	11.40 ± 0.95	11.35 ± 0.80	0.220
Preoperative APTT (s)	30.31 ± 1.80	29.89 ± 2.10	30.29 ± 2.18	30.19 ± 2.80	0.511
Intraoperative baseline					
Operation time (min)	63.8 ± 3.7	62.9 ± 4.1	64.2 ± 3.5	63.5 ± 3.9	0.085
Length of incision (cm)	10.1 ± 1.1	9.8 ± 1.2	10.3 ± 1.0	9.9 ± 1.3	0.029

TXA, tranexamic acid; FG, fluid gelatin; TXA + FG, tranexamic acid combined with fluid gelatin; BMI, body mass index; ASA, American Society of Anesthesiologists; PT, prothrombin time; APTT, activated partial thromboplastin time; L, left; R, right.

* Values are given as the mean ± standard deviation or as the number (percent).

### Perioperative blood loss

No significant differences were observed in preoperative day-1 HGB or HCT levels among the four groups. In contrast, on postoperative days 1 and 3, HGB and HCT levels in the TXA, FG, and TXA + FG groups were significantly higher than those in the control group. Among the intervention groups, the FG group exhibited lower HGB and HCT levels than the TXA group, whereas the combined TXA + FG group demonstrated the highest HGB and HCT levels across all groups.

With respect to perioperative blood loss, both TBL and HBL were significantly reduced in the three intervention groups compared with the control group. The FG group showed greater TBL and HBL than the TXA group, while the TXA + FG group achieved the lowest TBL and HBL among all groups. In contrast, no significant differences in VBL were observed among the four groups. Furthermore, the blood transfusion rate in the TXA + FG group was significantly lower than that in the control group, the TXA group, and the FG group. The only blood products transfused intraoperatively were red blood cells. Neither platelets nor plasma products were used (Table 2).

**Table 2:** Perioperative blood loss*.

Outcomes	Control group	TXA group	FG group	TXA + FG Group	*P*-value
Perioperative blood-related indicators					
Preoperative day 1					
HGB (g/L)	117.98 ± 5.66	118.46 ± 5.71	119.19 ± 5.35	118.70 ± 5.92	0.486
HCT (%)	37.11 ± 2.91	37.40 ± 3.21	37.24 ± 3.01	37.66 ± 3.14	0.593
Postoperative day 1 (POD1)					
HGB (g/L)	93.18 ± 5.82	101.48 ± 6.11^a^	96.35 ± 5.63^a^^,^^b^	105.22 ± 5.96^a^^,^^b^^,^^c^	<0.001
HCT (%)	29.74 ± 2.51	31.93 ± 2.20^a^	31.27 ± 2.26^a^	33.89 ± 2.63^a^^,^^b^^,^^c^	<0.001
Postoperative day 3 (POD3)					
HGB (g/L)	83.79 ± 5.52	94.43 ± 6.10^a^	91.21 ± 6.22^a^^,^^b^	99.78 ± 6.01^a^^,^^b^^,^^c^	<0.001
HCT (%)	28.45 ± 2.31	31.09 ± 2.13^a^	30.02 ± 2.21^a^^,^^b^	32.68 ± 2.25^a^^,^^b^^,^^c^	<0.001
Blood loss					
TBL (mL)	982.99 ± 428.81	732.50 ± 362.84^a^	817.19 ± 375.46^a^	550.68 ± 327.61^a^^,^^b^^,^^c^	<0.001
VBL (mL)	83.09 ± 21.04	86.02 ± 17.57	85.14 ± 20.13	81.69 ± 18.78	0.383
HBL (mL)	899.89 ± 430.41	650.27 ± 355.78^a^	737.30 ± 366.01^a^	474.03 ± 323.07^a^^,^^b^^,^^c^	<0.001
Perioperative transfusions (*n*, %)	19 (17.8%)	11 (11.8%)	16 (15.7%)	5 (4.7%)^a^^,^^b^^,^^c^	0.022
Type of blood product					
Packed red blood cells (unit)	19	11	16	5^a^^,^^b^^,^^c^	0.022
Platelet concentrates (unit)	0	0	0	0	
Fresh frozen plasma (mL)	0	0	0	0	
IV fluid amount					
Operation day (mL)	981.31 ± 422.96	1,021.51 ± 429.32	1,009.80 ± 404.07	985.85 ± 393.14	0.804
Postoperative day 1 (POD1)	519.62 ± 249.32	515.05 ± 248.89	589.22 ± 248.12	597.17 ± 246.68	>0.999
Postoperative day 2 (POD2)	210.28 ± 247.99	236.56 ± 250.99	264.71 ± 250.80	235.849 ± 250.79	0.999

TXA, tranexamic acid; FG, fluid gelatin; HGB, hemoglobin; HCT, hematocrit.

a Significantly different from the control group.

b Significantly different from the TXA group.

c Significantly different from the FG group.

* Values are given as the mean  ±  standard deviation or as the number (percent).

### Postoperative complications

There were 20 cases (18.9%) of deep vein thrombosis in the TXA + fluid gelatin group, compared with 34 cases (31.8%) in the control group, 23 cases (24.7%) in the TXA group, and 29 cases (28.4%) in the fluid gelatin group, but the differences were not statistically significant (*P* > 0.05). Pulmonary embolism and wound infection occurred in one patient in each of the TXA, the fluid gelatin, and the TXA + fluid gelatin groups, with no statistically significant differences (*P* > 0.05). One case of subcutaneous bruising was observed in the TXA + fluid gelatin group, which was lower than that in the control group, and the differences were statistically significant (*P* < 0.05), as shown in Table 3.

**Table 3:** Complications*.

Outcomes	Control group [*n* (%)]	TXA group [*n* (%)]	FG group [*n* (%)]	TXA + FG group [*n* (%)]	*P*-value
Thromboembolic events (no. of patients, %)	35 (32.7)	23 (24.7)	29 (28.4)	20 (18.9)	0.129
Deep vein thrombosis (DVT)	34 (31.8)	23 (24.7)	29 (28.4)	20 (18.9)	0.168
Pulmonary embolism (PE)	1 (0.9)	0 (0)	0 (0)	0 (0)	0.420
Wound complications (no. of patients, %)	9 (0.8)	2 (2.2)	2 (2.0)	1 (0.9)^a^	0.026
Infection	1 (0.9)	0 (0)	0 (0)	0 (0)	0.420
Bruising	8 (7.5)	2 (2.2)	2 (2.0)	1 (0.9)^a^	0.030

TXA, tranexamic acid; FG, fluid gelatin; HGB, hemoglobin; HCT, hematocrit.

a Significantly different from the control group.

* Values are given as the mean ± standard deviation or as the number (percent).

### Coagulation function

For coagulation-related parameters—including APTT, PT, D-dimer, and fibrinogen—levels remained consistent across all four groups (Table 4).

**Table 4:** Coagulation function*.

Outcomes	Control group	TXA group	FG group	TXA + FG group	*P*-value
Coagulation function					
APTT (s)	24.84 ± 1.59	25.00 ± 1.39	24.89 ± 1.42	24.99 ± 1.76	0.8492
PT (s)	10.93 ± 0.95	11.16 ± 1.13	10.96 ± 0.97	10.93 ± 0.96	0.3487
FDP (g/L)	3.398 ± 0.98	3.392 ± 1.05	3.567 ± 1.10	3.633 ± 0.94	0.2286
D-dimer (mg/L FEU)	2.472 ± 1.28	2.594 ± 1.22	2.512 ± 1.13	2.256 ± 1.26	0.2412

TXA, tranexamic acid; FG, fluid gelatin; HGB, hemoglobin; HCT, hematocrit.

* Values are given as the mean ± standard deviation or as the number (percent).

### Typical cases

**Figure 1:**
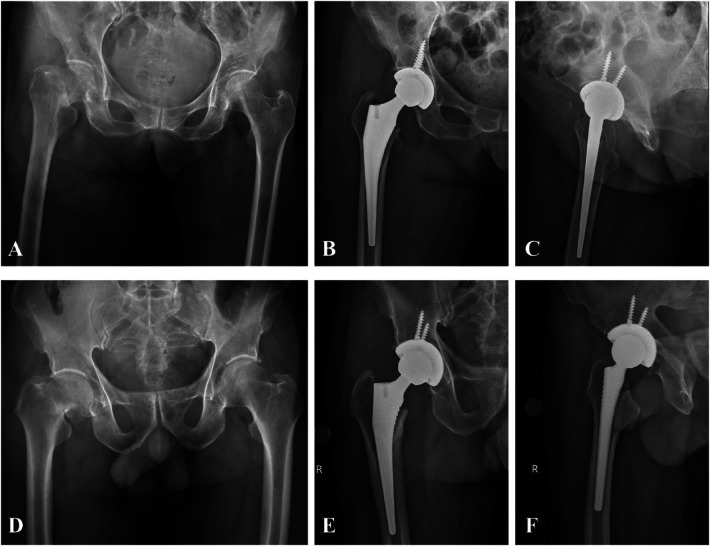
Perioperative DR results of typical cases. **(A)** Preoperative anteroposterior pelvis radiograph (Case 1). **(B)** Postoperative anteroposterior hip radiograph (Case 1). **(C)** Postoperative lateral hip radiograph (Case 1). **(D)** Preoperative anteroposterior pelvis radiograph (78M, right femoral neck fracture). **(E)** Postoperative anteroposterior hip radiograph (Case 2). **(F)** Postoperative lateral hip radiograph (Case 2).

Case 1: A 79-year-old female patient presented with a fracture of the right femoral neck, classified as Garden type III. She was treated with THA via the piriformis muscle approach. Preoperative DR (Digital Radiography) demonstrated a right femoral neck fracture (Figure 1A), postoperative anteroposterior (Figure 1B), and lateral (Figure 1C) DR.

Case 2: A 78-year-old male patient presented with a right femoral neck fracture, classified as Garden type III. He underwent THA via the piriformis muscle approach. Preoperative DR demonstrated a right femoral neck fracture (Figure 1D), postoperative anteroposterior (Figure 1E), and lateral (Figure 1F) DR.

## Discussion

With the intensification of population aging, the incidence of femoral neck fractures in the elderly has significantly increased. THA, as the main treatment method, significantly improves function and reduces disability rates. However, perioperative bleeding remains an important risk factor (16) affecting postoperative rehabilitation and prognosis. In elderly patients, the coexistence of systemic fibrinolytic activation induced by intraoperative trauma and local capillary bleeding can easily lead to an increase in latent bleeding, induce postoperative anemia, delay recovery, and even increase the risk (17) of postoperative complications. Therefore, constructing multimodal hemostasis intervention strategies has become the focus of clinical practice.

This study compared and analyzed the application effects of TXA and fluid gelatin in different pathways and found that the TXA group, fluid gelatin group, and combination group demonstrated significantly better outcomes than the control group in terms of total blood loss, hidden blood loss, and postoperative HGB and HCT indicators. The combination group achieved the best results, suggesting that systemic antifibrinolysis and local hemostasis mechanisms exert a synergistic effect. TXA—by inhibiting plasminogen conversion to block fibrin degradation and reduce systemic fibrinolytic activity—has been widely used in intraoperative and postoperative bleeding control during joint replacement surgery. Our results are consistent with previous literature (18), further supporting its clinical applicability and safety in elderly THA patients.

As mentioned previously, TXA is the most commonly used hemostatic agent in THA. Although local application of TXA in the surgical site can reduce perioperative blood loss (19), bleeding following THA remains a clinically significant issue that requires effective intervention. Moreover, when the dose of TXA is too high, its hemostatic effect plateaus and there is an increased risk of postoperative nausea and vomiting and a higher risk of deep venous thrombosis (20). Therefore, to further enhance the efficacy of TXA, we investigated whether local application of FG at the surgical site could reduce perioperative blood loss during THA.

FG is an absorbable hemostatic colloid material with a unique fluid consistency, which enables uniform distribution around bleeding sites, maximizes contact with injured tissues, and thereby lays a foundation for efficient hemorrhage control (21). FG exerts its hemostatic effects through multiple synergistic mechanisms: Firstly, it directly blocks bleeding points on the wound surface after local injection (22). Secondly, it activates the natural coagulation cascade and promotes platelet aggregation to accelerate the formation of stable clots (23). Finally, its matrix structure provides physical tamponade and acts as a scaffold for platelet adhesion and aggregation, further enhancing clot stability and bleeding cessation (21).

Notably, FG possesses excellent biocompatibility with inherent absorbability. When properly applied, it is completely degraded and absorbed in the human body within 4–6 weeks, avoiding long-term tissue irritation or foreign body reactions (24). Due to these integrated advantages (effective hemostasis, favorable fluidity, and reliable absorbability), FG has been widely used in neurosurgery, spinal surgery, orthopedics, and other surgical disciplines to achieve reliable intraoperative hemostasis, particularly in scenarios such as joint capsule bleeding and complex surgical field hemorrhage (9, 21–25).

In this study, the fluid gelatin group demonstrated improvement over the control group in terms of the extent of HGB reduction after surgery, hidden blood loss, and the incidence of subcutaneous congestion, verifying its positive role in local hemostasis. The combined use of TXA and fluid gelatin also had the lowest blood transfusion rate, at 4.7%, significantly lower than the other groups (*P* < 0.05), demonstrating the clinical potential of synergistic hemostasis in optimizing blood management.

With regard to postoperative complications, we found that the combination of FG and TXA did not increase the risk of thromboembolic events, which are the most common complications of TXA use. As FG functions as a scaffold material, it enables local hemostasis through physical adsorption and intermolecular interactions, such as ionic and hydrogen bonding. Consequently, this combination can achieve enhanced hemostatic efficacy during the perioperative period under the same dose of TXA, thereby avoiding the need for high-dose TXA administration and its associated complications while achieving superior hemostasis. In addition, the incidence of bruising around the wound in the FG + TXA group was significantly lower than that in the control group, but there was no difference compared with the other groups. These findings further support the combined application of FG and TXA, which has been shown to effectively reduce perioperative blood loss.

As blood loss increases, a patient's circulating blood volume tends to decrease. However, concurrent fluid shifts into the intravascular compartment and perioperative fluid administration help maintain circulating volume, albeit with progressive hemodilution—characterized by declining hematocrit—resulting in isovolemic hemodilution. Therefore, cases involving large fluid infusions were excluded from this study to minimize confounding effects. Moreover, perioperative intravenous fluid administration was recorded for all patients; however, such fluid management did not significantly contribute to hemodilution in this cohort (15).

The strength of this study lies in the enhanced comparability of data based on a single surgical approach (minimally invasive piriformis approach) and a unified perioperative management process. The introduction of a local fluid gelatin injection intervention during the operation fills the gap in previous studies that focused only on the use of the TXA system, highlighting the value of dual-mechanism synergistic hemostasis. However, this study also has limitations, such as being a single-center retrospective study with potential selection bias. The short follow-up period made it impossible to assess long-term safety and functional outcomes. The adaptability and long-term efficacy of this strategy need to be verified through multicenter, prospective randomized controlled studies in the future.

To sum up, intravenous tranexamic acid combined with intra-articular local injection of fluid gelatin can synergistically reduce total perioperative blood loss and hidden blood loss in elderly patients with femoral neck fractures undergoing THA, reduce the need for postoperative blood transfusion, and not increase the risk of postoperative thromboembolism and wound complications. This combination may further enhance blood management efficiency in MIS-THA and may be applied to other THA approaches.

## Data Availability

The raw data supporting the conclusions of this article will be made available by the corresponding author on reasonable request.
